# Optimized protocol for combined PALM-dSTORM imaging

**DOI:** 10.1038/s41598-018-27059-z

**Published:** 2018-06-08

**Authors:** O. Glushonkov, E. Réal, E. Boutant, Y. Mély, P. Didier

**Affiliations:** Laboratoire de Bioimagerie et Pathologies, UMR 7021 CNRS, Université de Strasbourg, 67000 Strasbourg, France

## Abstract

Multi-colour super-resolution localization microscopy is an efficient technique to study a variety of intracellular processes, including protein-protein interactions. This technique requires specific labels that display transition between fluorescent and non-fluorescent states under given conditions. For the most commonly used label types, photoactivatable fluorescent proteins and organic fluorophores, these conditions are different, making experiments that combine both labels difficult. Here, we demonstrate that changing the standard imaging buffer of thiols/oxygen scavenging system, used for organic fluorophores, to the commercial mounting medium Vectashield increased the number of photons emitted by the fluorescent protein mEos2 and enhanced the photoconversion rate between its green and red forms. In addition, the photophysical properties of organic fluorophores remained unaltered with respect to the standard imaging buffer. The use of Vectashield together with our optimized protocol for correction of sample drift and chromatic aberrations enabled us to perform two-colour 3D super-resolution imaging of the nucleolus and resolve its three compartments.

## Introduction

Fluorescence microscopy has become an invaluable tool for cellular studies, both on structural and functional levels. A large number of labelling methods enable the specific attachment of fluorophores and fluorescent reporters to structures of interest in fixed and living cells^1^. Moreover, this non-invasive approach allows spatiotemporal observation of living specimens making optical far-field fluorescence microscopy a method of choice for imaging a large range of biological processes. In addition, the use of different fluorophores offers the possibility to visualize simultaneously different structures and to quantitatively characterize protein-protein interactions with the help of approaches such as Förster Resonance Energy Transfer coupled to Fluorescence Lifetime Imaging Microscopy (FRET-FLIM)^2^ or Fluorescence Cross Correlation Spectroscopy (FCCS)^3^. However, in their conventional implementations, these methods are restricted by the diffraction phenomenon that limits their spatial resolution to about 200 nm in the lateral plane and 500 nm along the axial direction.

A breakthrough has been achieved by the development of high-resolution microscopy, that provides sub-diffraction resolution and introduces the extremely promising era of “nanoscopy”^4^. Both scanning and wide-field nanoscopy approaches were developed^5^. In the case of scanning imaging, point spread function (PSF) engineering methods allow to reduce the size of the emitting fluorescent volume. On the other hand, individual emitters are localized with high precision in wide-field nanoscopy techniques. Random switching schemes are exploited to determine the position of a single emitter much more precisely than the optical resolution of the instrument. The schemes are generally based on photoswitchable fluorescent proteins (PALM, photoactivation localization microscopy)^6,7^ or organic dyes^8,9^ (dSTORM, direct stochastic optical reconstruction microscopy; GSDIM, ground-state depletion followed by individual molecule return).

Under appropriate irradiance flux density, one-colour experiments are quite simple to perform^10,11^. In the case of two-colour experiments, one has first to determine the appropriate fluorophore pairs that can stain the system of interest while minimizing the spectral overlap. Photoactivable fluorescent proteins (PA-FP) and fluorescently labelled antibodies (F-Ab) can be used to perform two-colour super-resolution experiments. PA-FPs allow imaging overexpressed proteins (plasmid transfection) or modified endogenous proteins (CRISPR/Cas9) with a non-invasive one-to-one tagging but display a limited brightness compared to F-Ab that can target endogenous proteins. As two-colour super-resolution experiments with two PA-FPs are not straightforward to implement because of the limited number of available PA-FP pairs^12,13^, a PA-FP can be combined with a F-Ab to demonstrate the co-localisation of two proteins with an improved spatial resolution. However, implementing such a two-colour super-resolution experiment is challenging due to the different parameters that need to be controlled simultaneously: (I) two lasers are required to control the ON-OFF conversion (for PA-FP) and generate the highest photon count rate within the shortest time interval; (II) appropriate imaging buffer composition is needed to ensure proper ON-OFF transition rate for both labels; (III) mechanical drift and chromatic aberrations between two channels need to be quantified and corrected.

In this work, we optimized all these parameters to perform 2D and 3D two-colour super-resolution experiments using a photoswitchable FP (mEos2)^14^ in combination with an Ab labelled with Alexa Fluor 647 (A647-Ab). The nucleolus was chosen as a model system to apply this optimized protocol. This non enveloped nuclear compartment is subdivided into three sub-domains that are all associated with specific steps of ribosome biogenesis: fibrillar centres (FCs) – transcription of rDNA; dense fibrillar component (DFC) – pre-rRNA processing and modification (methylation, pseudouridylation); and granular component (GC) – assembly of ribosomal subunits^15^. These sub-domains have been spatially resolved by means of electron microscopy^15^, but not with classical optical imaging. To resolve these sub-nucleolar domains by high resolution PALM/dSTORM microscopy, we used fibrillarin (Fib), nucleophosmin (NPM) and RPA (RPA194 – the largest subunit of RNA-polymerase-I) proteins as specific markers of the DFC, GC and FC subdomains, respectively^16^. These proteins were either overexpressed in fusion to the protein mEos2 or their endogenous forms were immunostained with specific A647 labelled antibodies. Two imaging buffers were compared to determine the most suitable one for simultaneous use of mEos2-FP and A647-Ab. The localization precision and the number of detected single emitters per frame were used as a benchmark to compare the two buffers. To obtain a perfect reconstruction, we also optimized the drift correction method by using quantum dots as fiducial markers to allow correction in both channels. Finally, we applied a procedure to minimize and correct the chromatic aberrations inherent to any optical microscope in order to generate 2D and 3D images of the nucleoli.

## Results and Discussion

### Confocal imaging

**Figure 1:**
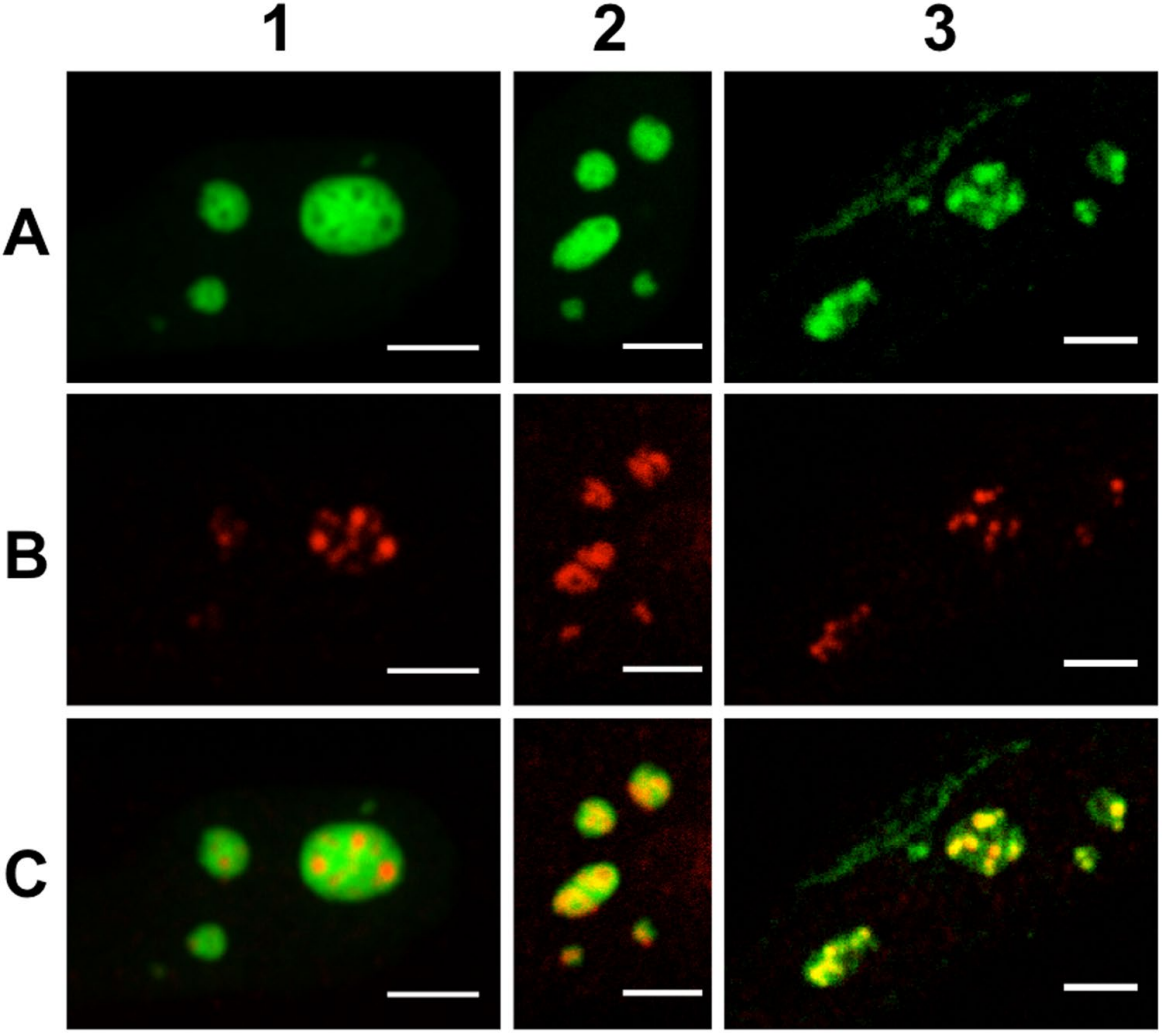
Confocal microscopy imaging of nucleolar sub-domains in HeLa cells. Column 1: NPM protein fused to the fluorescent protein eGFP was overexpressed after cell transient transfection in order to image the nucleoli granular component (**A1**). Endogenous RPA was immunolabelled with A647-Ab (**B1**). The merged images evidenced the specificity of the labelling strategy (**C1**). Column 2: eGFP-NPM localization is displayed in panel A2. Endogenous Fib was immunolabelled with A647-Ab (**B2**). The merged images evidenced the spatial overlap between the two domains (**C2**). Column 3: Endogenous Fib was immunolabelled with A555-Ab (**A3**). Endogenous RPA was immunolabelled with A647-Ab (**B3**). The merged images evidenced the spatial overlap between the two domains (**C3**). Scale bar: 5 µm.

Confocal imaging was first used to assess our labelling strategies and in particular the immunostaining specificity. In order to visualize the nucleolar sub-domains, HeLa cells were transiently transfected to express the eGFP-NPM fusion protein and were simultaneously immunostained with A647 (or Alexa Fluor 555, A555)-labelled antibodies targeting Fib or RPA proteins. As depicted in the first column of Fig. 1, eGFP-NPM (A1) can be imaged simultaneously with RPA (A647, B1) and confocal imaging allows to clearly distinguish these two compartments. The absence of co-localisation in this case clearly supports the specificity of our labelling strategies. In experiments performed with eGFP-NPM (Fig. 1A2) and Fib (A647, Fig. 1B2) or with Fib (A555, Fig. 1A3) and RPA (A647, Fig. 1C3), there is a significant overlap between the two channels making impossible to spatially discriminate the two labelled domains with confocal microscopy. Nevertheless, the fluorescence intensities distributions differ for the three labelled proteins, strongly suggesting that as expected, the three proteins have different localizations in the nucleoli as it was evidenced by electron microscopy experiments^15^. The observed spatial overlaps between the fluorescence distributions (NPM/Fib and Fib/RPA) confirm that the diffraction-limited resolution of the confocal microscope is not sufficient to discriminate the different sub-domains. To overcome this limitation, we next performed super-resolution experiments with cells expressing a photoswitchable FP (mEos2) fused to NPM and immunostained with an A647-Ab targeting Fib or RPA.

### Buffer selection

**Figure 2:**
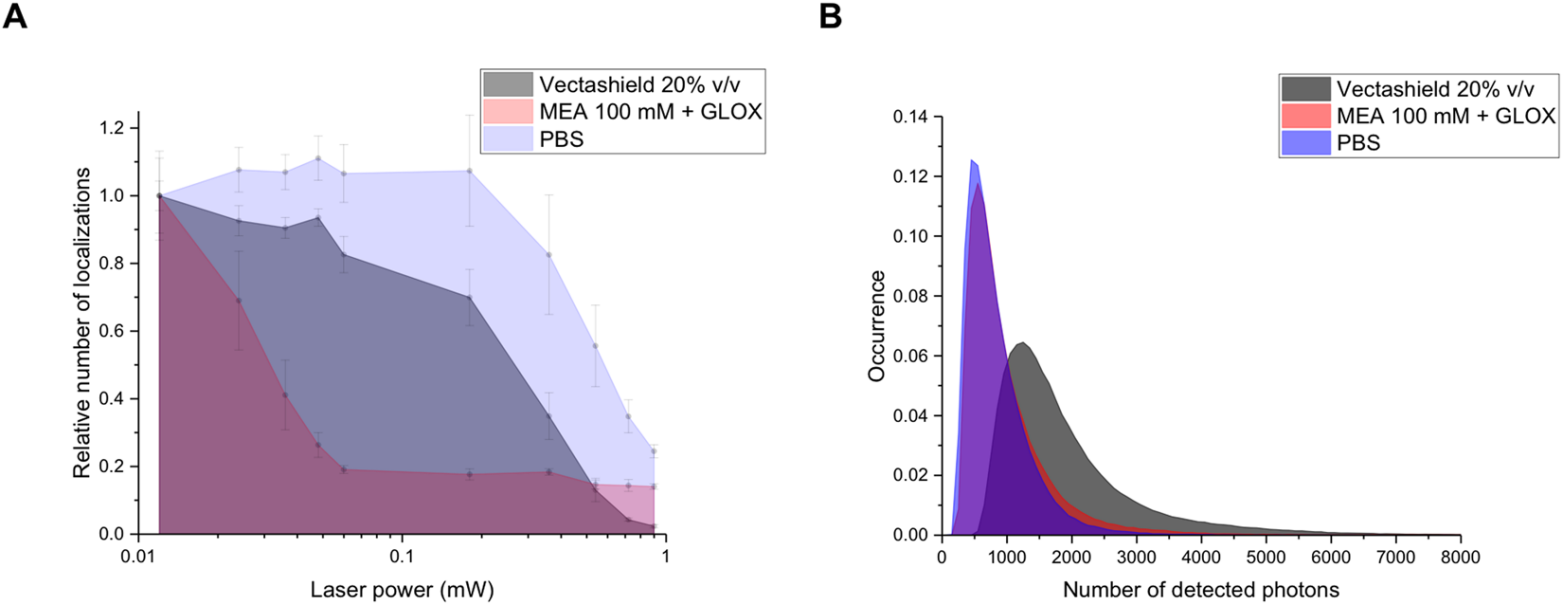
Comparison of the photophysical properties of overexpressed mEos2 in aqueous buffer (PBS), in the standard imaging buffer of thiols with oxygen scavenging system (MEA 100 mM + GLOX) and in the Vectashield mounting medium. (**A**) Relative number of localizations as a function of the UV laser power. A single cell was first imaged for 30 s with the 561 nm laser to bleach already activated mEos2. The same cell was then imaged using the 405 and 561 nm lasers for 30 s. Each point corresponds to number of detected localizations over 30 s for different UV laser power. All the points were normalized by the number of localizations measured during the first 30 s. (**B**) Number of detected photons per molecule per frame (integration time: 30 ms). The mode values are 500, 550 and 1250 photons for PBS, MEA + GLOX and Vectashield respectively. The median number of photons are 711, 793 and 1602 photons for PBS, MEA + GLOX and Vectashield respectively. These results (**A** and **B**) were obtained from the average of 5 different cells for each condition.

In a first series of experiments, a comparison between different imaging buffers was performed to determine the optimal conditions ensuring appropriate ON-OFF conversion for both labels (mEos2 and A647). To this aim, single colour imaging was performed with cells expressing a plasmid coding for mEos2.The standard imaging buffer of thiols/oxygen scavenging system^11^ (i.e. MEA 100 mM + GLOX, see Buffers section in Materials and Methods) was found to generate a high ON-OFF switching rate of A647. In contrast, a decrease in the number of photoactivations of mEos2 was observed over time with this imaging buffer (Fig. 2A), likely as a result of a reaction between the thiols and the fluorescent protein chromophore. To reduce the effect of the imaging buffer on mEos2, it is possible to wash the sample with PBS buffer before starting a new acquisition. However, this step might modify the position of the sample on the microscope stage and damage the cells making this approach difficult to implement.

As an alternative to the conventional thiol-containing buffers, we tested the Vectashield medium that is used in microscopy as an antifading mounting medium and that has been recently shown to induce blinking of organic fluorophores^17^. The main advantage of this ready-to-use medium relies on its pH stability. In contrast, the pH of the standard imaging buffer is changing over time and might impact the photophysical properties of the fluorescent labels^18,19^. However, the Vectashield medium displays an absorption band in the UV range that generates some background with the high laser powers used in localization microscopy. Although this background limits the range of fluorophores that can be used to the orange-red range of the visible spectrum, it can be reduced by diluting Vectashield in glycerol. Several pairs of dyes, such as A555 with A647 were shown to work in two-colour experiments with Vectashield^17^.

Figure 2A represents the relative number of localizations measured during 30 s for different UV laser powers in three different imaging buffers (PBS, thiol + oxygen scavenging system and Vectashield). As the concentration of the overexpressed proteins varies from cell to cell, modifying thus the absolute number of localizations per frame, we monitor the relative changes in the number of localizations between the three imaging buffers. To do so, the measurements were performed by using the following protocol: we first imaged a single cell during 30 s with the 561 nm laser to bleach the already activated mEos2 proteins. Then we imaged the sample with the 405 and 561 nm lasers for 30 s with a fixed UV laser power (0.012 mW). The measured number of localizations over 30 s gives thus the total number of localizations (N_1_). The UV laser power was gradually increased and the total number of localizations (N_i_) was determined for the same ROI over 30 s after each change of UV laser power. The obtained number of localizations (which corresponds to the number of detected photoactivations for a 30 s time interval) for each UV laser power was normalized by N_1_ (N_i_/N_1_) to give the relative changes of the number of localizations. This measurement was repeated on five cells for the three imaging buffers allowing to provide a more reliable comparison. Interestingly, the relative number of localizations (Fig. 2A) was lower for the thiols/oxygen scavenging buffer compared to Vectashield (except for power greater than 0.5 mW). Using appropriate UV laser power (in our case <0.5 mW) it will be possible to obtain an increased total number of localizations for the Vectashield medium. One can note that aqueous buffer (PBS) provides higher photoactivation rate compared to Vectashield and thiols/oxygen scavenging buffers. In addition, we determined the distributions of the number of blinks after photoactivation and ON time of mEos2 for the three imaging buffers. As reported in Fig. S1 the obtained results evidenced that the buffer composition has no noticeable effects on these parameters. Finally, we measured the brightness of mEos2 for the three imaging buffers (Fig. 2B). The results clearly indicate that, in the Vectashield medium, the number of photons emitted by mEos2 per frame (exposure time 30 ms) was twice higher than in the standard imaging buffer (and PBS) while the A647 brightness was similar for both imaging buffer (Fig. S2). We did not see any difference in the blinking rate of A647 with respect to the standard imaging buffer. As the localization precision depends, in first approximation, on the number of detected photons (${\rm{\Delta }}x\approx \frac{\sigma }{\sqrt{N}}$, where *Δx* is the localization precision, *σ* is the standard deviation of the Gaussian fit and *N* is the number of detected photons), mEos2 will thus be more accurately localized in the Vectashield medium^20^. Vectashield appears thus as an improved medium as compared to the standard imaging buffer to simultaneously monitor mEos2 and A647 localization.

Fluorescent proteins and organic fluorophores that are usually used in localization microscopy require different buffers for efficient blinking and high photon yield. Georgieva *et al*. show that the standard thiols/oxygen scavenging imaging buffer with a lower concentration of the reducing agent can be used for simultaneous use of these labels^21^. However, the main drawback of this multi-component buffer relies in the limited duration of the enzymatic oxygen scavenging reaction (one hour after mixing the components) which limits the duration of the experiment and results in buffer acidification^17,18^.

### Drift correction

In a next step, our aim was to optimize the corrections of the mechanical drift of the sample. These corrections are usually performed with fiducial markers, such as TetraSpek fluospheres, gold nanoparticles or quantum dots^6,8^ or with the help of cross-correlation image analysis^22,23^. Two conditions need to be fulfilled to use fiducial emitters: (I) their brightness should be similar to that of fluorescent labels in order to work with the same exposure time and camera gain (to avoid saturation); and (II) they must be photostable during the acquisition time. Indeed, high and constant brightness throughout the acquisition of thousands of frames is required for high and constant localization precision.

TetraSpek fluospheres (T7279, Invitrogen, 100 nm) and gold nanoparticles (753688, Sigma-Aldrich, 100 nm) were added during cell growth and incorporated via endocytosis. They remained immobilized after cell fixation with 4% PFA. Despite being very bright, TetraSpek beads are prone to rapid photobleaching under high excitation power. In addition, the camera gain used for amplifying the signal of a single mEos2 protein or A647 dye leads to the saturation of the signal of the fluorescent beads, making challenging their use for drift correction. In the case of gold nanoparticles, the signal results from the scattering of the excitation beam by the nanoparticles allowing their use for multicolour imaging. Under our experimental conditions (excitation intensity: 2 kW/cm^2^), the gold nanoparticle signal was suddenly disappearing before the end of the experiment. Moreover, the signal detected for the gold nanoparticles was not high enough to allow a precise correction of the mechanical drift. A similar behaviour was observed with gold nanoparticles incorporated after cell fixation.

Finally, we investigated the possibility to use quantum dots (QD, Qdot 655 Streptavidin Conjugate - Q10121MP, Invitrogen, 15–20 nm) in our experiments. When incorporated via endocytosis in living cells, we observed that their emission intensity was continuously decreasing, probably due to the low pH in endosomes. In contrast, when QDs were added directly after fixation during incubation with antibodies, the intensity of the emitted signal was constant over the entire acquisition. Moreover, the emitted intensity of a single QD was of the same order as that of mEos2 and A647 on the two detection channels. The drift correction was thus performed with the same precision for mEos2 and A647. Importantly, we did not observe any photobleaching of the intensity of the signal emitted by the QDs during the acquisition time in these conditions. However, QDs blink, so that among the stack of images, QDs disappear in some of them. Hence, an interpolation must be performed to retrieve the full trajectory. It is worth to note that the concentration of the QDs solution prepared in a 1.5 mL Eppendorf tube is changing over time (more than a month), even if the tube is sonicated and vortexed before the experiment. We recommend thus to prepare a fresh solution at least once in a month by diluting the purchased 1 µM stock solution of Qdot 655 in MilliQ water to achieve a final concentration of ∼1 nM. Prepared solutions must be tested in cells at different dilutions to get the desired number of QDs per field of view. All super-resolution images displayed in this work were generated using QDs for drift correction. The properties of the fiducial markers measured in Vectashield are summarized in the following table. The measured intensities of the emitted signal obtained for a TetraSpek bead, a gold nanoparticle and a quantum dot are displayed in Fig. S3.

**Table:** 

	**Incorporated before fixation**		**Incorporated after fixation**	
	**Photostability**	**Brightness**	**Photostability**	**Brightness**
TetraSpek	−	+++	−	+++
Gold Nanoparticle	−	−	−	−
QD	−	+	+	+

### Correction of chromatic aberration

**Figure 3:**
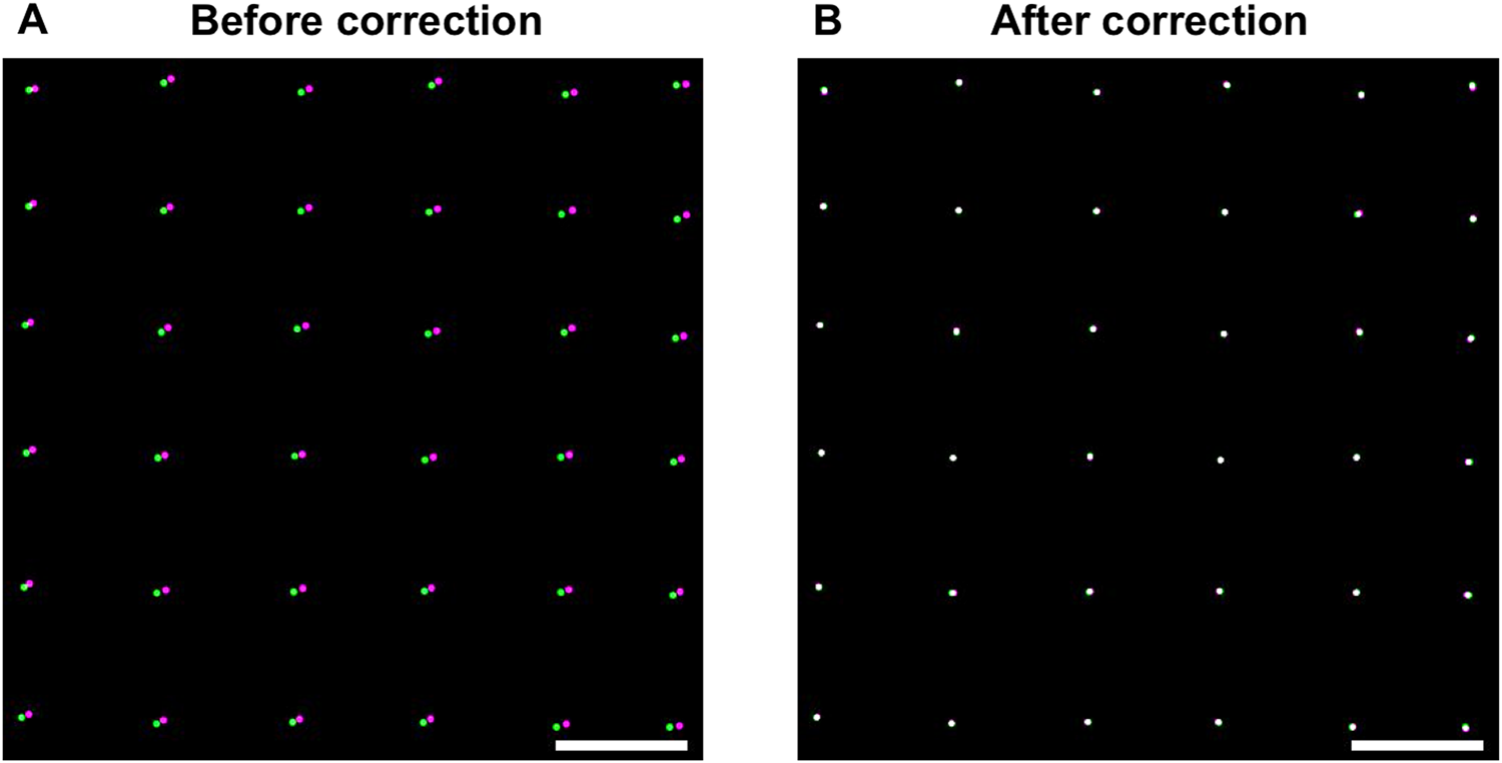
Correction of lateral chromatic aberrations. The images were obtained by performing a raster scan with a single TetraSpek bead (a detailed protocol is given in the SI). The final reconstructed image was obtained by localizing the bead on each individual frame with Thunder STORM ImageJ plugin for both channels (rendering with a PSF of 20 nm). The displayed images correspond to a zoom of one corner of the camera field of view (27.3 × 27.3 µm^2^). (**A**) The largest distance between green and magenta spots is about 100 nm before correction. (**B**) Two-colour image obtained after correction with the UnwarpJ plugin. Scale bar: 1 µm.

An additional step is requested to correct for the residual chromatic aberration in our two-colour imaging experiments. The correction was performed with the previously mentioned TetraSpek fluospheres excited with a low laser power that does not induce any significant bleaching during measurements. The Gemini system from Hamamatsu was used to split the camera’s chip in two spatially separated spectral windows. We first performed a raster scan of a single bead, using the microscope translation stage, to record a reference image and determined, for each frame, its localization with the help of Thunder Storm ImageJ plugin^24^ (a detailed protocol for the acquisition of the reference image is given in the SI). The residual chromatic aberration, which results in a spatial shift between the two channels (100 nm at the edge of the field in Fig. 3A), was corrected using the UnwarpJ plugin^25^. The algorithm computes the correction matrix required for a perfect superposition of the two channels. In Fig. 3, we reported the colocalization image corresponding to the corner of the field of view (27.3 × 27.3 µm^2^) before and after correction.

The residual chromatic aberration between the two channels will depend on the bandpass filters used for imaging. The emission spectra of the reference sample (TetraSpek beads) and the fluorophores used for imaging must then overlap. Axial chromatic aberration may also take place. To evaluate it, we performed a z-scan on a single TetraSpek bead and fitted the PSFs of both green and red channels with a 2D Gaussian function. By plotting the dependence of the standard deviation as a function of the z position, we obtained a curve with a minimum where beads are in focus. The minima for both channels must be at the same z-coordinate (Fig. S4) to have minimal axial aberration.

### 2D PALM/dSTORM imaging of nucleolar sub-domains

After having optimized our measurement conditions and corrections, we next imaged the labelled sub-domains of nucleoli. HeLa cells expressing mEos2-NPM fusion protein were immunostained with A647-Ab directed against Fib or RPA, stored at +4 °C and finally mounted on microscope slides on the day of imaging. Before mounting, the cells were washed with Milli-Q water to remove salts. Each time, we followed a pre-imaging routine, in which the Gemini system was adjusted in order to align the two channels by imaging in transmitted light the reference target provided with the Gemini system. Next, TetraSpek beads deposited on a glass coverslip were used to optimize the PSF with the help of adaptive optics^26^ to minimize various aberrations (spherical, astigmatism, coma, trefoil) introduced by optical elements, and the refractive index mismatch between the objective and the immersion medium. This optimization is important because aberrations affect the quality of the PSF and reduce the number of detected photons, which results in lower localization precision and lower resolution. Finally, the same reference sample was used to perform a raster scan in order to generate the correction matrix required to correct the chromatic aberration between the two channels.

**Figure 4:**
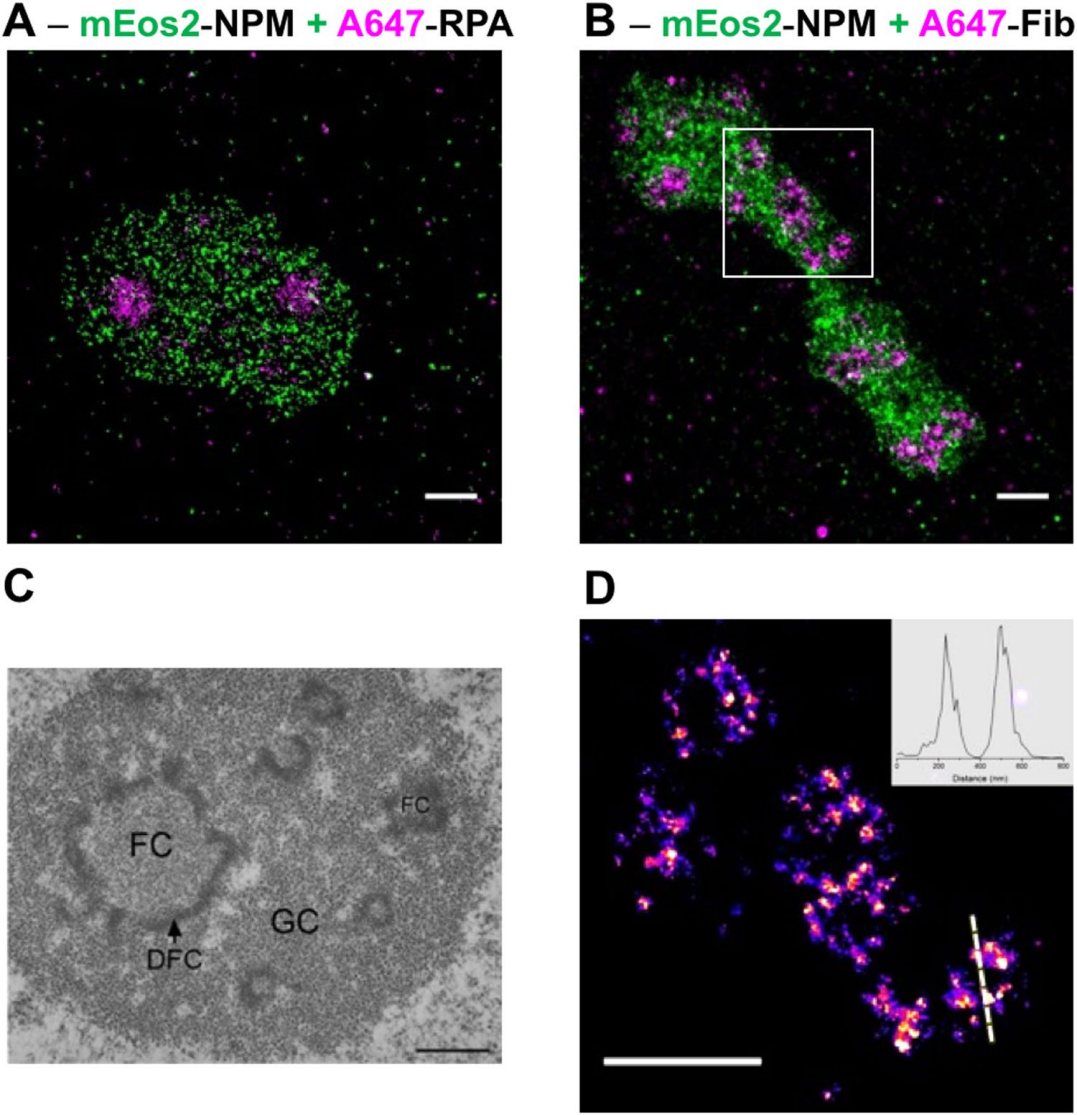
Two-colour super-resolution imaging of nucleolar sub-domains. (**A**) HeLa cells granular component (GC) was imaged with the help of NPM protein fused to mEos2 (green). The fibrillar centres (FCs) were imaged by immunostaining RPA proteins with A647-Ab (magenta). (**B**) HeLa cells GC was visualized together with the dense fibrillar component (DFC), by using mEos2-NPM in combination with Fib proteins immunostained by A647-Ab (magenta). (**C**) Nucleolar organization of human HeLa cell observed by electron microscopy. Reprinted with permission of the author and the editor (permission is granted to Macmillan Publishers Ltd, part of Springer Nature)^31^. (**D**) Ring-like structure of DFC revealed by super-resolution localization microscopy (zoom of the white ROI in **B**). The inner diameter (FWHM = 160 nm) and the thickness (FWHM = 75 nm) of the ring were measured from the cross section displayed in inset. All super-resolution images were obtained from a 20000 images stack as described in the main text. Scale bars: 1 µm.

To image the sample, we performed a sequential acquisition. We first imaged the red channel (A647) to observe Fib and RPA localizations, then both 405 nm and 561 nm lasers were used for the acquisition of the green channel (to monitor the photoconverted form of mEos2-NPM). This sequential acquisition was needed because of the high staining efficiency with A647-Ab, which induces a saturation of the camera when exciting the sample with all laser sources simultaneously. Between five to ten thousand frames were recorded per channel. Thanks to the strong brightness of A647 and its fast ON-OFF transition, it was possible to reduce the exposure time to 16 ms, resulting in shorter acquisition times. For the fluorescent protein mEos2, the exposure time was set to 30 ms. Next, the data were treated and analysed with the Thunder Storm plugin of ImageJ (see parameters in Materials and Methods section). Rendered images (Fig. 4) were corrected for the drift (QDs) and then for the chromatic aberrations (reference sample of TetraSpek beads).

In Fig. 4, we reported the two-colour super-resolution images of HeLa cells nucleoli. The granular component visualized through the emission of mEos2-NPM was co-imaged with the DFC (Fig. 4A) or FCs (Fig. 4B), immunostained with A647-Abs directed against Fib and RPA, respectively. The average localization precision was about 15 and 10 nm for the green and red channel, respectively. As observed by using confocal microscopy, the domains stained by NPM and RPA are mutually exclusive (Fig. 4A) confirming the specificity of our labelling strategy. In the case of GC and DFCs, the images (Fig. 4B) obtained using super-resolution microscopy did not show any colocalization confirming the gain in spatial resolution with respect to confocal imaging. Moreover, the ring like structures revealed by electron microscopy (Fig. 4C) could also be observed by super-resolution microscopy for some nucleoli (Fig. 4D). The improved spatial resolution of the images is further illustrated with the cross-section displayed in Fig. 4D from which it is possible to measure the inner diameter (FWHM = 160 nm) and the thickness (FWHM = 75 nm) of the ring-like domain.

### 3D imaging of nucleolus

While working in 2D, the spatial information along the z-axis is lost because of the projection onto the camera plane. As the size of the PSF does not change significantly over the depth of field (Fig. S5), different parts of the object that appear in focus may in fact localize at different depths within the sample. For instance, a hollow sphere will be visualized as a full disk if the diameter of this sphere is less than the depth of field (<400 nm). To obtain spatial information along the z-axis, we next used the 3D imaging capability on our setup, based on adaptive optics.

**Figure 5:**
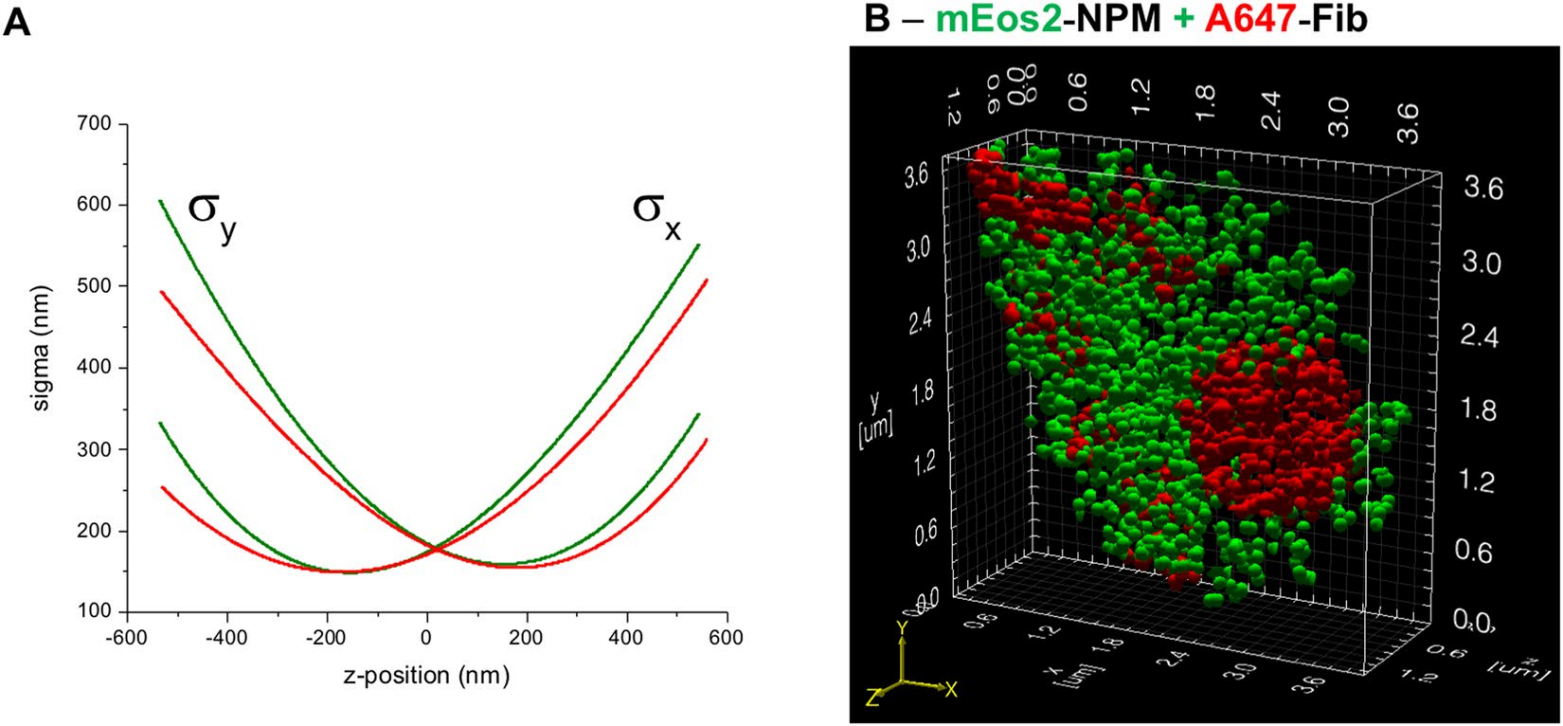
Two-colours 3D imaging of nucleolar sub-compartments. (**A**) The calibration curves for the green and red channels were obtained by performing a z-scan on a single TetraSpek bead. The sigma values (x and y) correspond to the width of the 2D Gaussian fit used to localize the bead position. The difference between the two sets of curves are higher for |**Δz**| > **200** **nm**. Therefore, the two channels must be treated individually. (**B**) Two-colour 3D image of the granular and dense fibrillar sub-domains visualized by using mEos2-NPM and A647-Ab directed against fibrillarin. Stacks (10000 images for each colour) were analysed with Thunder Storm to obtain the localization coordinates (x, y and z) from which it was possible to make a 3D surface rendering with the help of ViSP software.

Among the different methods allowing to perform 3D imaging^23,27,28^, adaptive optics can be used to introduce astigmatism in order to create an asymmetric PSF with respect to the circle of least confusion. Therefore, the shape and orientation of this PSF depends on the z-position of the dye. By fitting the PSF with a two-dimensional Gaussian function, two sigma values are obtained from which the z-position of the fluorophore can be determined using a calibration curve (see Fig. 5A). To generate the calibration curve, TetraSpek beads were immobilized on a glass coverslip and we performed a z-scan over a 1.4 µm depth, while exciting with a 561 nm laser at low power. The analysis was done with the help of the Thunder STORM ImageJ plugin and the obtained calibration curves are reported in Fig. 5A.

Next, we performed two-colour imaging on cells expressing mEos2-NPM and immunostained with A647-Ab directed against Fib. The two channels were analysed individually using the Thunder STORM ImageJ plugin and the calibration curves to retrieve the x, y and z coordinates of each single emitter. A table containing the coordinates and the number of detected photons was next imported in the ViSP software in order to obtain a surface rendering for a better representation^29^. As depicted in Fig. 5B, it is possible to discriminate in 3D the granular and the dense fibrillar components with a lateral localization precision of 15 and 10 nm for the green and the red channels respectively. The axial localization precision depends on the number of detected photons and z-coordinate^30^. This value is high for |**Δz**| > **50** **nm**, where σ_x_ ≈ σ_y_ (including the error of σ determination) and decreases for higher z values. In our case, the highest axial localization precision was about 20 nm for both channels. Among the different modalities of electron microscopy^31^, it is possible to obtain a 3D rendering, however the associated protocol is much more restrictive. Indeed, to generate 3D images, an ultramicrotome must be used to remove very thin layer (50–200 nm) on the fixed sample before the acquisition of the 2D image. This process is repeated until the whole sample volume is reconstructed. In addition, sample preparation and selection of fixation agent are critical for ultrastructural preservation and image acquisition. Finally, the contrast obtained in electron microscopy is less specific than the one obtained in fluorescence microscopy. Despite being less spatially resolved, 3D two-colour super-resolution imaging appears as an excellent alternative to 3D electron microscopy.

## Conclusions

In this study, we demonstrated that the Vectashield mounting medium (Vector laboratories) does not only improve the photophysical properties of the fluorescent protein mEos2 but is also much easier to use than the standard imaging buffer of thiols/oxygen scavenging system. Indeed, one just needs to dilute the Vectashield in glycerol to reduce the background in the green channel caused by its autofluorescence. Later, buffer can be stored at +4 °C for several weeks and reused again for other super-resolution experiments. In addition, the number of photons emitted by mEos2 is increased while keeping constant the A647 brightness. The mounting medium Vectashield gives thus a higher level of freedom for the selection of labelling strategy, which is important to address various biological problems. The high refractive index of the buffer also provides a better index matching for 3D imaging than water-based buffers. A protocol for multicolour drift correction based on the use of quantum dots was also presented. The main advantage of these fiducial emitters relies on their brightness, which is similar to that of the fluorescent labels used for imaging under our excitation conditions. The drift correction is thus performed with a similar localization precision. Moreover, quantum dots allow the experimentalist to avoid detector saturation that is for instance observed with TetraSpek beads. The residual chromatic aberrations between the detection channels was corrected *a posteriori* with the help of a reference image obtained with TetraSpek beads. All these optimizations allowed us to perform 2D and 3D two-colour super-resolution imaging of the nucleolar sub-domains that were spatially resolved for the first time, to our knowledge, with the help of fluorescence imaging.

## Materials and Methods

### Plasmid constructs

The peGFP-NPM construct was kindly provided by Xin Wang (plasmid # 17578, Addgene).

The mEos2-NPM construct was obtained by replacing the eGFP cDNA in the peGFP-C1-GW vector corresponding to the Gateway® cloning version of peGFP-C1 (Clontech) in order to obtain the vector pmEOs2-C1-GW. To do so, the mEos2 cDNA was inserted between NheI and XhoI restriction sites of a digested peGFP-C1 GW. The cDNA coding for NPM was thus PCR amplify with primers harbouring the attB recombination sequences and cloned using the Gateway technology in pmEOs2-C1-GW to obtain the fusion mEos2-NPM. The integrity of all plasmid constructs was assessed by DNA sequencing (GATC Biotech, Germany).

### Cell culture and transfection

HeLa cells were cultured in DMEM (1 g/L D-Glucose, [−] Phenol Red; Gibco by LifeTechnologies) supplemented with 10% Foetal Bovine Serum (S1810–500, Dutscher), 1% of Pen-Strep solution (100 units of Potassium Penicillin and 100 μg of Streptomycin Sulfate per 1 mL of culture media – final concentration; DE17-602E, Lonza, BioWhitaker) and 1% L-Glutamine (2 mM; BE17-605E, Lonza, BioWhitaker) at 37 °C with 5% CO_2_. Cells were transiently transfected with different plasmids on the next day after seeding using jetPEI transfection reagent (PolyPlus Transfection) following the supplier’s protocol −2 µL of jetPEI for 1 µg of plasmid DNA.

### Immunofluorescent labelling

Cells were seeded on round cover-glasses 18 mm in diameter (62407–063, VWR) at a density of 75000 cells per well of a 12-well plate (131024C, ClearLine). Prior to seeding, cover-glasses were washed once with 70% ethanol and then three times with DPBS (Buffered Saline 0.0095M (PO_4_), Lonza, BioWhitaker). For imaging, cells were fixed in 4% PFA (15710, Electron Microscopy Science) (diluted in DPBS) for 12 min at 37 °C and then, permeabilized with 0.2% Triton X-100 (X100–500ML, Sigma) (diluted in DPBS) during 10 min at room temperature. These steps were followed by 3 washes with DPBS for 5 min. Non-specific sites were blocked using 1% BSA (sc-2323, ChemCruz) during 1 h. Primary antibodies @NPM (7H10B0) (NBP1-47354, Novus biological), @Fibrillarin (38F3) (MA3-16771, Invitrogen) and @RPA194 (F-6) (sc-46699, Santa Cruz) were diluted 500 and 50 times respectively in 1% BSA. To save the stock of antibodies, drops of 50 µL were deposited on a parafilm and then cover-glasses were placed on the top for 1 h. Cells were washed again three times with DPBS. The steps of dilution (750 to 1000 times for both), deposition and incubation were repeated with the secondary antibodies F(ab′)2-goat @mouse-A647 (A21237, Invitrogen) and goat @mouse-A555 (A21424, Invitrogen). QDs (Qdot 655 Streptavidin Conjugate - Q10121MP, Invitrogen) were added into the solution with antibodies at 2 pM. The QDs are used to correct for the sample drift that occurs during a long acquisition. Incubation with this mixture was done for 45 min at room temperature. After final washing, samples were covered with an aluminium foil and kept at +4 °C before imaging (one-week maximum).

### Buffers

The standard switching medium for organic fluorophores is composed of TN buffer (50 mM Tris (pH 8.0) and 10 mM NaCl), an oxygen scavenging system (0.5 mg/mL glucose oxidase (G7141-50KU, Sigma), 40 µg/mL catalase (C40-500MG, Sigma) and 10% (w/v) glucose) and 100 mM β-mercaptoethylamine (MEA; 30070-10G, Sigma). Buffer was freshly prepared on the day of the experiment.

The second buffer was prepared by mixing Vectashield (H-1000, Vector Laboratories) in TRIS-Glycerol (5% v/v TRIS 1 M pH 8 in Glycerol) to achieve a final concentration of 20% Vectashield. A 10 µL drop of this solution was deposited on a microscope slide, then a cover-glass (our sample) was rinsed in MilliQ water to remove salts and placed on the top of that drop. After 10 min at room temperature, enough to evaporate water from the surface of the cover-glass, the sample was sealed with a dental cement (Picodent). The sample is ready for imaging and can be stored at +4 °C for several days and reused again later.

### Setup and imaging

Confocal microscopy imaging was performed on a Leica SP2 microscope equipped with 63X oil-immersion objective (1.2 NA). For confocal imaging, Fib and RPA were immunolabeled with Abs labelled with Alexa Fluor 555 and Alexa Fluor 647, and NPM was expressed as a fusion protein with the fluorescent protein eGFP. The fluorescent labels were excited at 635 nm (Alexa Fluor 647), 561 nm (Alexa Fluor 555) and 488 nm (eGFP) and their emission was collected (650–750 nm; 570–630 nm, 500–540 nm) by a PMT detector.

Super-resolution localization microscopy imaging was performed on a home-built setup based on a Nikon Eclipse Ti microscope with 100x 1.49 NA oil-immersion objective. The laser lines at 488 nm, 561 nm and 642 nm (Oxxius) were used for excitation of mEos2, A555, A647, and the 405 nm laser was used for photoconversion of mEos2. Laser power during the experiments was set to 50 mW for 561 nm and 642 nm lasers, that results in 2 kW/cm^2^ excitation intensity, and 0.5 to 120 W/cm^2^ for 405 nm laser. Laser lines were co-aligned into a single beam using single-band dichroic mirrors (Semrock). An acousto-optic tunable filter (AOTF; Opto-Electronic) was used to switch between lasers (shutter) and change the laser power. Emission from the sample was spectrally filtered with the help of a multi-band dichroic mirror (405/488/561/635 nm, lasers BrightLine quad-edge super-resolution laser dichroic beamsplitter: Di03-R405/488/561/635-t1-25x36, Semrock) and notch filters (561 nm and 642 nm StopLine single-notch filters: NF03-561E-25 and NF03-642E-25, Semrock; in order to remove the scattered laser light), and then was imaged on an EM-CCD camera from Hamamatsu (ImagEM). An additional lens was used to obtain a final magnification of 150X corresponding to a pixel size of 106.67 nm. For two-colour experiments, the signal on the camera chip was split into two channels using an image splitting optics Gemini (Hamamatsu) with a dichroic mirror (640 nm edge BrightLine single-edge imaging-flat dichroic beamsplitter: FF640-FDi01-25x36, Semrock) and long-pass filters (561 nm and 647 nm EdgeBasic long-pass filters: BLP02-561R-25 and BLP01-647R-25) inside. Each time before imaging the sample, the point spread function was optimized, and residual optical aberrations (spherical, coma, …) were minimized using adaptive optics (Imagine Optics) with the help of TetraSpek bead^32^. The reference image with TetraSpek beads (ThermoFischer) was acquired to correct the lateral shift and chromatic aberrations (UnwarpJ plugin, ImageJ) between the two channels. Z-stabilization was ensured by the perfect focus system (PFS, Nikon Eclipse Ti) on the microscope.

### Data analysis

A stack of 10000–20000 images of 512 * 256 pixels (two channels together) was analysed with the Thunder STORM plugin in ImageJ. The following parameters were used to find and fit the signal of each particle: image filtering – Difference-of-Gaussians filter (sigma 1 = 1.0 and sigma 2 = 1.6); approximate localization of molecules – Local maximum (peak intensity threshold: std(Wave.F1), connectivity: 8-neighbourhood); sub-pixel localization of molecules – Integrated Gaussian (fitting radius: 4 px, fitting method: Least squares, initial sigma: 1.3 px). Results were filtered by sigma and localization precision values: 120 nm < sigma < 180 nm (see Fig. 4 in SI), precision < 25 nm. Drift correction: with fiducial markers Max distance – 100 nm, Min marker visibility ratio – 0.15, Trajectory smoothing – 0.03. Reference images with TetraSpek beads were analysed with the UnwarpJ plugin of ImageJ in order to calculate the elastic deformations and create the transformation matrix, which will be applied to rendered images for correction of chromatic aberration.

### Data availability

The raw data of the results presented in the paper are available upon request to the corresponding author.

## Electronic supplementary material

Supplementary information
