# Supplementary material for: Optimized protocol for combined PALM-dSTORM imaging
Source: Sci Rep. 2018 Jun 8;8:8749. doi: 10.1038/s41598-018-27059-z (PMC5993819; doi:10.1038/s41598-018-27059-z)
Supplement: Supplementary file 1 — Supplementary information [file 41598_2018_27059_MOESM1_ESM.pdf]

Supplementary Information

Optimized protocol for combined PALM-dSTORM imaging

Oleksandr Glushonkov, Eleonore Real, Emmanuel Boutant, Yves Mely, Pascal Didier*

Laboratoire de Bioimagerie et Pathologies, UMR 7021 CNRS, Université de Strasbourg, 67000 Strasbourg, France

**corresponding author: pascal.didier@unistra.fr*

Correction of the chromatic aberration.

In order to obtain a reference image for the correction of the chromatic aberration, we immobilized TetraSpek beads on a glass coverslip. The sample was next imaged under low excitation power at 561 nm to prevent photobleaching of the beads. By using this excitation wavelength, it was possible to excite two different dyes encapsulated in the bead. In that case, the single bead appears simultaneously in the green and red channel of the camera mounted after the Gemini module (Frame 0 on the scheme). As the chromatic aberration depends on the position in the field of view, we used the translation stage of the microscope to move the bead at specific positions in the field of view.

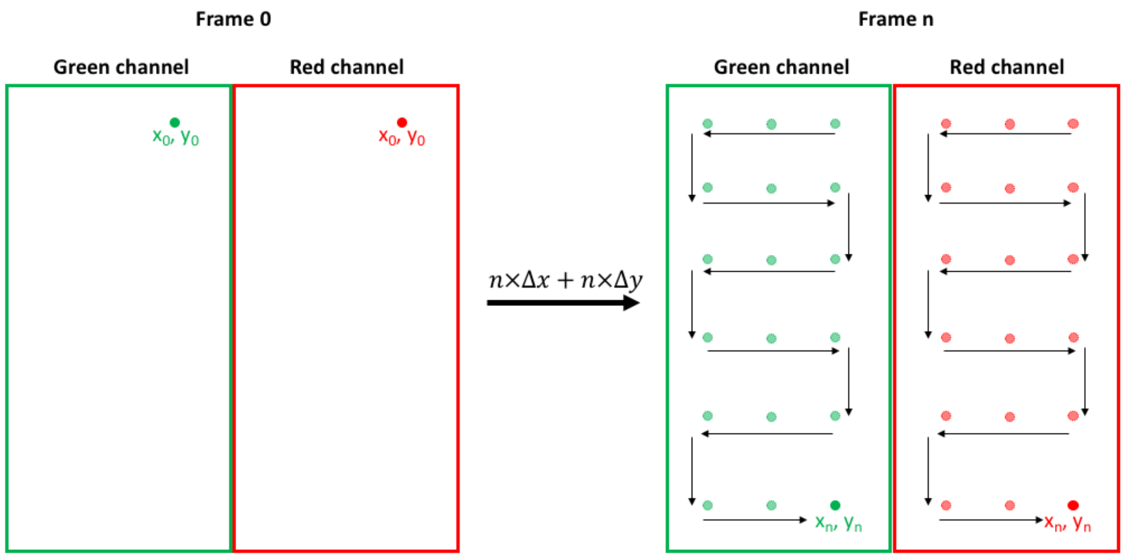

Thunder STORM plugin was then used to localize the position of the bead. For all positions we recorded an image with the bead appearing in both channels. By summing all the retrieved localization (with a PSF rendering of 20 nm), it was possible to obtain a reference image in which the same bead is placed in a periodic manner on different positions within the field of view (Frame n on the scheme). Without any chromatic aberration, a perfect superposition of the two channels could be obtained after cropping the green and the red channels. However, as depicted in Figure 3A such procedure does not allow to obtain a perfect superposition of the two channels because of the position depend chromatic aberration. Nevertheless, as the green and red spots originates from the same bead, an algorithm can be used to correct *a posteriori* the position depend chromatic aberration. To do so, we used the UnwarpJ plugin of ImageJ which is able to generate a field dependent deformation matrix by superposing the bead position on the green and the red channels. The obtained deformation matrix can be later used to correct the chromatic aberration on the images obtained with the biological sample. We recommend to record a reference image every day to account for the day to day change of the optical alignment.

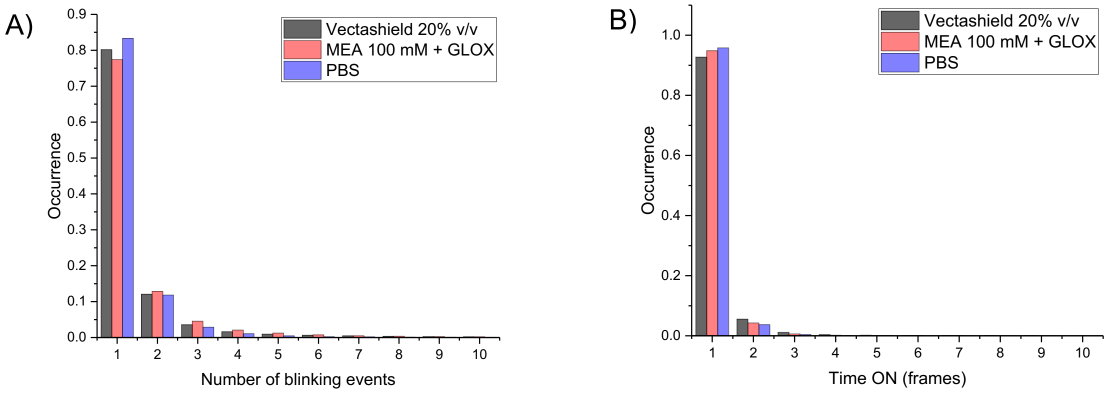

Figure S1. Comparison of the photophysical properties of overexpressed mEos2 in aqueous buffer (PBS), in the standard imaging buffer of thiols with oxygen scavenging system (MEA 100 mM + GLOX) and in the Vectashield mounting medium. A) Distributions of the number of blinks after photoactivation. B) Distributions of the ON time measured for mEos2 after photoactivation.

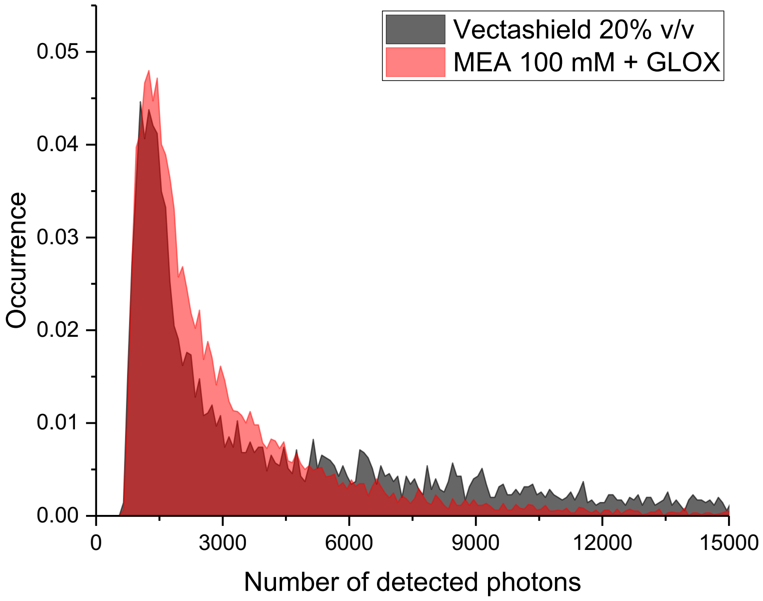

Figure S2. Photon number distributions of A647-Ab imaged in the standard Imaging buffer of thiols/oxygen scavenging system and in the Vectashield mounting medium (integration time: 16 ms). The mode values are 1240 and 1250 photons for MEA+GLOX and Vectashield respectively. The median number of photons values are 2079 and 2580 photons for MEA+GLOX and Vectashield respectively.

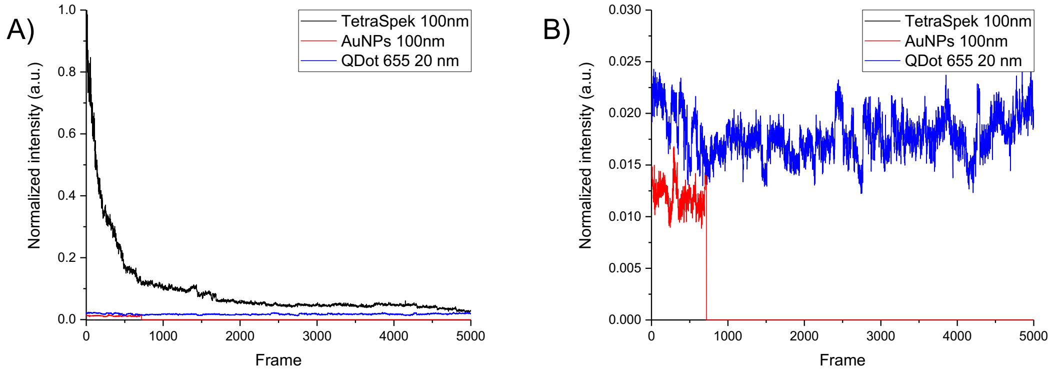

Figure S3. Comparison of the intensities of the signal emitted by single fiducial markers. The TetraSpek beads, gold nanoparticles (Au NPs) and quantum dots (QDs) were immobilized on a glass coverslip using Vectashield as mounting medium. At high laser power that is common for localization microscopy experiments, TetraSpek beads display very high intensity leading to the saturation of the camera with the EM gain used to maximize the number of detected photons per single fluorophore (panel A). In addition, under such high excitation regime, TetraSpek are prone to photobleach. Under our experimental conditions, the intensity of the signal emitted by single Au NP disappeared after few hundred images while the intensity of the signal emitted by the QD could be monitored over the entire acquisition (panel B).

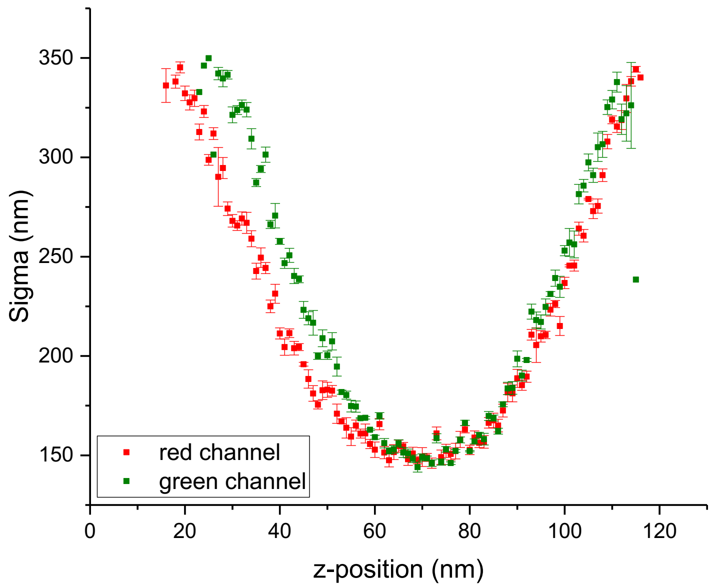

Figure S4. Axial chromatic aberration. A Z-scan of TetraSpek beads was performed with steps of 10 nm. The localizations of the beads in two different channels were fitted separately using a 2D Gaussian model. The standard deviation is reported as a function of the z-coordinate for each channel. The minima of both curves must co-localize to allow imaging of the same sample region in both channels.

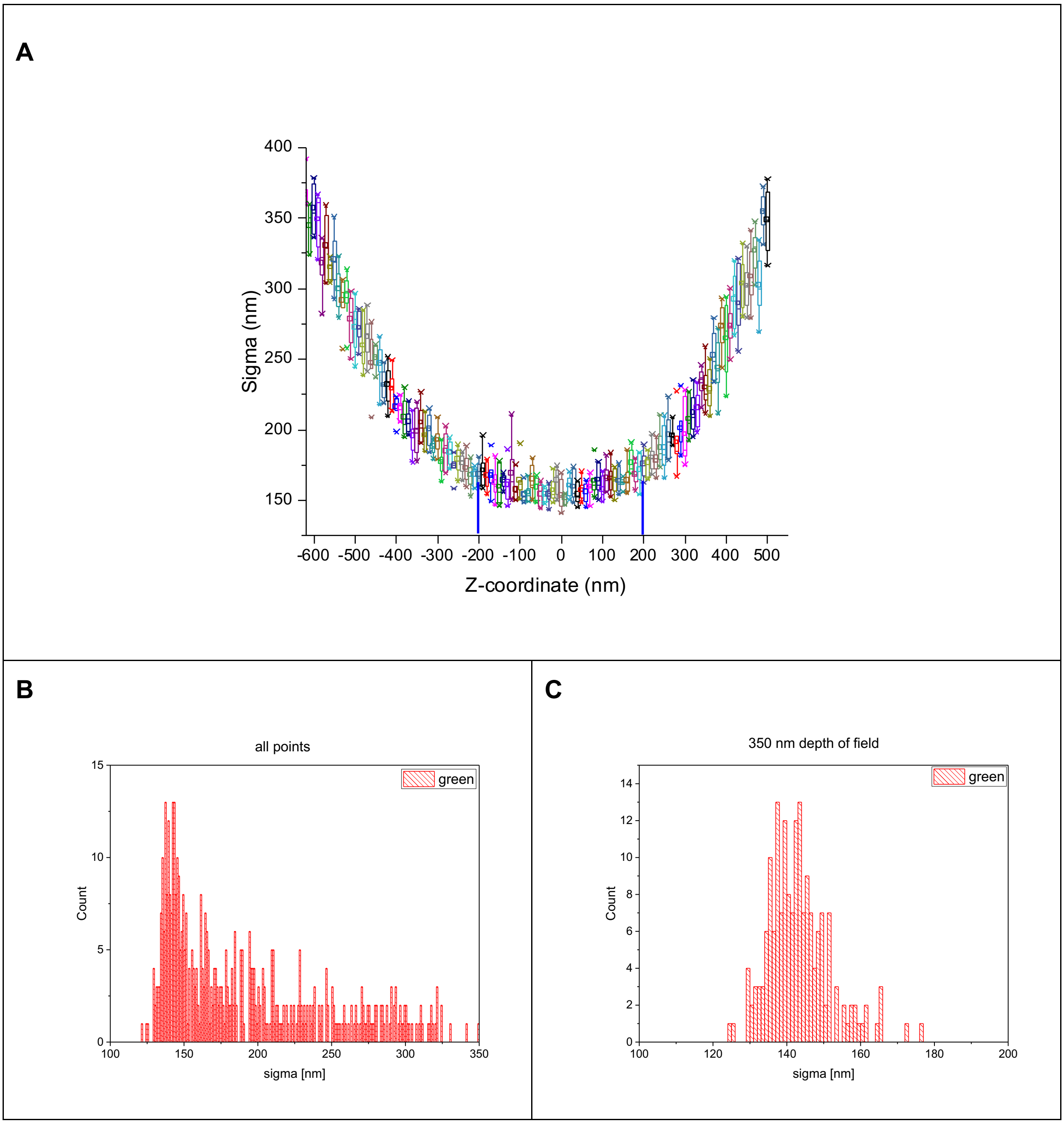

Figure S5. Measured sigma values as a function of the z position. (A) z-scan with 10 nm steps was performed on TetraSpek beads. At the minimum of the curve, where beads are in focus, the value of sigma (standard deviation of the Gaussian function) is around 150 nm. This value is almost constant over a range of 400 nm (blue bars). (B) Sigma values distribution obtained from the Gaussian fit of mEos2 localizations. The tail of the distribution (high sigma values) is associated to detection artefacts (e.g. two particles within diffraction-limited region or particles out of focus). (C) Sigma values distribution after filtering with a threshold: 120 nm < σ < 180 nm (150 nm ± 20%).
